# Arhgef5 Binds α-Dystrobrevin 1 and Regulates Neuromuscular Junction Integrity

**DOI:** 10.3389/fnmol.2020.00104

**Published:** 2020-06-10

**Authors:** Krzysztof M. Bernadzki, Patrycja Daszczuk, Katarzyna O. Rojek, Marcin Pęziński, Marta Gawor, Bhola S. Pradhan, Teresa de Cicco, Monika Bijata, Krystian Bijata, Jakub Włodarczyk, Tomasz J. Prószyński, Paweł Niewiadomski

**Affiliations:** ^1^Laboratory of Synaptogenesis, Nencki Institute of Experimental Biology, Warsaw, Poland; ^2^Institute of Physical Chemistry, Polish Academy of Sciences, Warsaw, Poland; ^3^Laboratory of Cell Biophysics, Nencki Institute of Experimental Biology, Warsaw, Poland; ^4^Łukasiewicz Research Network – PORT Polish Center for Technology Development, Wrocław, Poland; ^5^Laboratory of Molecular and Cellular Signaling, Centre of New Technologies, University of Warsaw, Warsaw, Poland

**Keywords:** neuromuscular junction, dystrophin-glycoprotein complex, Arhgef5, Rho GEFs, Rho-family GTPases, muscle development, neuromuscular disease

## Abstract

The neuromuscular junctions (NMJs) connect muscle fibers with motor neurons and enable the coordinated contraction of skeletal muscles. The dystrophin-associated glycoprotein complex (DGC) is an essential component of the postsynaptic machinery of the NMJ and is important for the maintenance of NMJ structural integrity. To identify novel proteins that are important for NMJ organization, we performed a mass spectrometry-based screen for interactors of α-dystrobrevin 1 (aDB1), one of the components of the DGC. The guanidine nucleotide exchange factor (GEF) Arhgef5 was found to be one of the aDB1 binding partners that is recruited to Tyr-713 in a phospho-dependent manner. We show here that Arhgef5 localizes to the NMJ and that its genetic depletion in the muscle causes the fragmentation of the synapses in conditional knockout mice. Arhgef5 loss *in vivo* is associated with a reduction in the levels of active GTP-bound RhoA and Cdc42 GTPases, highlighting the importance of actin dynamics regulation for the maintenance of NMJ integrity.

## Introduction

The neuromuscular junction (NMJ) is an essential interface between the central nervous system and a skeletal muscle fiber. It consists of the presynaptic axonal terminals, terminal Schwann cells that play a role in its maintenance and regeneration, and the postsynaptic machinery localized on the surface of the myofiber. The postsynaptic part of the NMJ is composed of an intricate array of protein complexes that facilitate high concentration of neurotransmitter receptors for acetylcholine (AChR) and allows for generation of muscle action potential. The postsynaptic machinery is linked to the extracellular matrix through membrane-embedded receptors. Many components of the postsynaptic apparatus provide a platform for the recruitment of the intracellular signal transduction machinery. Others anchor the NMJ to the cytoskeletal scaffold, and particularly to the F-actin network.

During postnatal development, the postsynaptic part of the NMJ is transformed from a simple plaque-like form into an elaborate, branched structure that resembles a pretzel. The molecular basis underlying this transformation is poorly understood. Studies on *in vitro* models (cultured muscle cells) suggested that the remodeling of the postsynaptic machinery is accomplished through actin-based organelles known as podosomes (Proszynski et al., 2009; Proszynski and Sanes, 2013; Bernadzki et al., 2014), which create gaps between AChR-rich areas. In cultured myotubes, the inhibition of podosome formation leads to altered distribution of AChR receptors in postsynaptic clusters (Proszynski et al., 2009). However, the function of podosomes in NMJ development *in vivo* has not been elucidated.

Apart from podosomes, the actin cytoskeleton is important for the formation and maintenance of postsynaptic AChR assemblies. AChR are anchored to F-actin (Mitsui et al., 2000) and actin dynamics drives AChR trafficking and clustering (Dai et al., 2000; Lee et al., 2009). Specifically, the regulation of actin cytoskeleton by Rho family GTPases appears to be involved in postsynaptic AChR clustering (Luo et al., 2002; Weston et al., 2003; Shi et al., 2010). The mechanisms of recruitment and regulation of Rho GTPases at the NMJ are poorly understood.

The dystrophin-glycoprotein complex (DGC) is a major muscle receptor for extracellular laminins and an important component of the postsynaptic NMJ machinery (Ervasti and Campbell, 1991; Nishimune et al., 2008; Gawor and Prószyński, 2018). The core of the DGC complex consist of dystrophin, syntrophin, α-dystroglycan, β-dystroglycan, the sarcoglycan complex, sarcospan, and α-dystrobrevin (Nakamori and Takahashi, 2011; Aittaleb et al., 2017; Belhasan and Akaaboune, 2020). The dysfunction of the DGC core components leads to myopathies in humans, including Duchenne muscular dystrophy, a disease characterized by progressive damage and impaired regeneration of skeletal muscles (Campbell, 1995). DGC core components can recruit additional, peripherally associated proteins. For instance the cytoplasmic protein α-dystrobrevin 1 (aDB1) is believed to be an adaptor for recruitment of various signaling molecules (Oh et al., 2012; Gingras et al., 2016; Gawor and Prószyński, 2018). The loss of aDB1 in mice results in abnormal NMJ morphology and impaired maturation of the postsynaptic apparatus (Grady et al., 2003, 2000, 1999). In humans, aDB1 mutations cause congenital heart disease with left ventricular non-compaction (Ichida et al., 2001). The function of aDB1 is dependent at least in part on its phosphorylation by tyrosine kinases (Grady et al., 2003; Schmidt et al., 2011; Gingras et al., 2016). To identify the mechanisms of the regulation of NMJ maturation by aDB1, we have previously searched for proteins that interact with aDB1 in a phosphorylation-dependent manner using a protein pull-down assay followed by mass spectrometry (Gingras et al., 2016). One of the proteins that we thus identified as an aDB1 interactor was Arhgef5. Arhgef5 is a guanidine nucleotide exchange factor (GEF) for the small GTPases from the Rho family and is involved in the regulation of actin dynamics (Xie et al., 2005). Interestingly, Arhgef5, which also interacts with another aDB1-binding protein α-catulin (Lyssand et al., 2010; Gingras et al., 2016) was shown to be pivotal for the Src-dependent formation of podosomes (Kuroiwa et al., 2011). We therefore hypothesized that Arhgef5 may cooperate with aDB1 and α-catulin to regulate the maturation and stability of the NMJ postsynaptic machinery by altering the dynamics of the actin cytoskeleton via Rho-family GTPases. Here, we show that Arhgef5 localizes at the NMJ *in vivo* and concentrates at the postsynaptic machinery. Loss of Arhgef5 in mouse skeletal muscles results in NMJ defects characterized by increased fragmentation of the postsynaptic apparatus, an effect that may be attributed to the abnormal function of the GTPases RhoA and Cdc42.

## Results

### Arhgef5 Binds to Phosphorylated aDB1s

**FIGURE 1:**
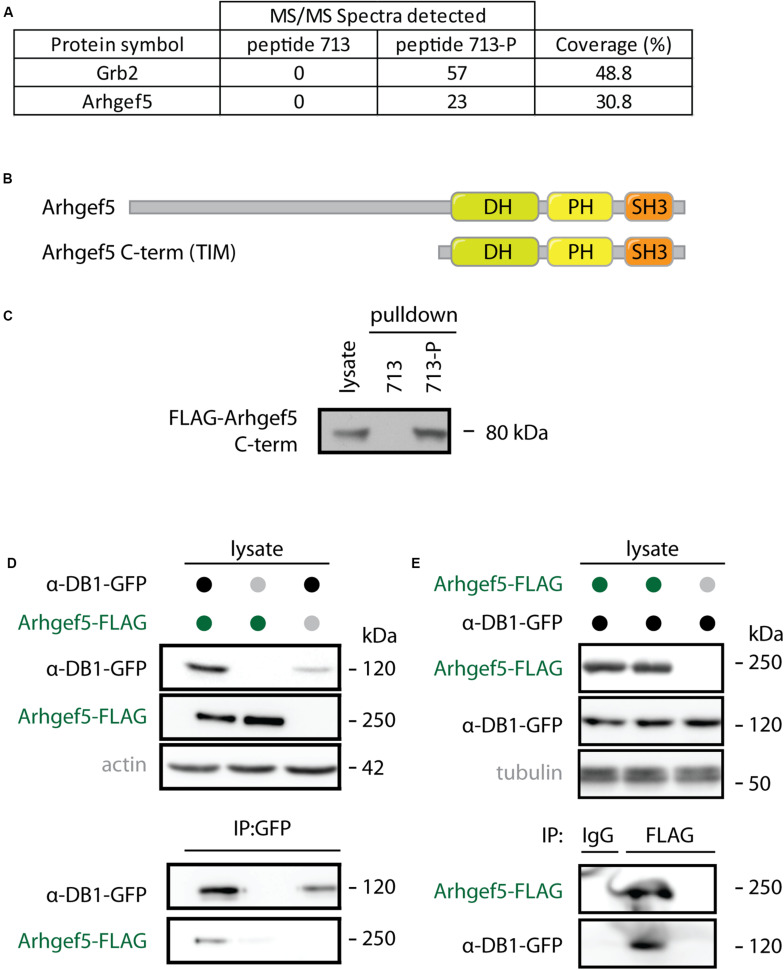
Arhgef5 binds to aDB1. **(A)** Lysates from C2C12 cells were incubated with beads coupled to either the unphosphorylated (713) or the phosphorylated (713-P) variant of aDB1 peptide containing the Y713 residue. Eluates from the beads were analyzed using mass spectrometry. Number of peptides from Grb2 and Arhgef5 identified in each eluate and percent peptide coverage of the full-length protein are shown. Data shown is a reanalysis of supplementary information from Gingras et al. (2016). **(B)** Domain architecture of Arhgef5 and its shorter C-terminal isoform TIM (only detected in humans). **(C)** FLAG-Arhgef5 C-terminal isoform was expressed in HEK293T cells and lysates were incubated with beads coupled to peptide 713 or 713-P. Eluates from the beads were analyzed by immunoblot. **(D,E)** FLAG-tagged Arhgef5 and GFP-tagged aDB1 were coexpressed in HEK293T cells, as indicated. **(D)** GFP-aDB1 was immunoprecipitated from the lysates using anti-GFP-coupled beads and eluates were analyzed by immunoblot. **(E)** FLAG-Arhgef5 was immunoprecipitated from the lysates using anti-FLAG- or rabbit IgG-coupled beads and eluates were analyzed by immunoblot.

Arhgef5 was originally identified in our unbiased mass spectrometry-based screen for interaction partners of the phosphorylated form of aDB1. Arhgef5 was one of the top proteins from myotube extracts that specifically bind the aDB1-derived phosphopeptide TQPEDGN**pY** ENESVRQ (Y713-P; corresponding to phosphorylated tyrosine 713 of aDB1) but not to its unphosphorylated control peptide TQPEDGN**Y** ENESVRQ (Y713) (Figure 1A). Arhgef5 has a typical domain structure of Rho GEFs: it contains a Dbl homology (DH), a pleckstrin homology (PH), and a Src homology 3 (SH3) domain (Figure 1B). Additionally, it contains an N-terminal domain that has several proline-rich motifs (Kuroiwa et al., 2011). In humans, in addition to the full-length protein, a shorter isoform called TIM lacking the N-terminal domain is expressed, but this isoform has not been identified in mice. Using western blot, we confirmed that the C-terminal domain of Arhgef5 binds the Y713-P, but not the Y713 peptide (Figure 1C). We also independently showed that full-length Arhgef5 binds to full-length aDB1, as evidenced by co-immunoprecipitation of overexpressed proteins (Figures 1D,E).

### Arhgef5 Localizes to AChR-Rich Domains of the Neuromuscular Junction

**FIGURE 2:**
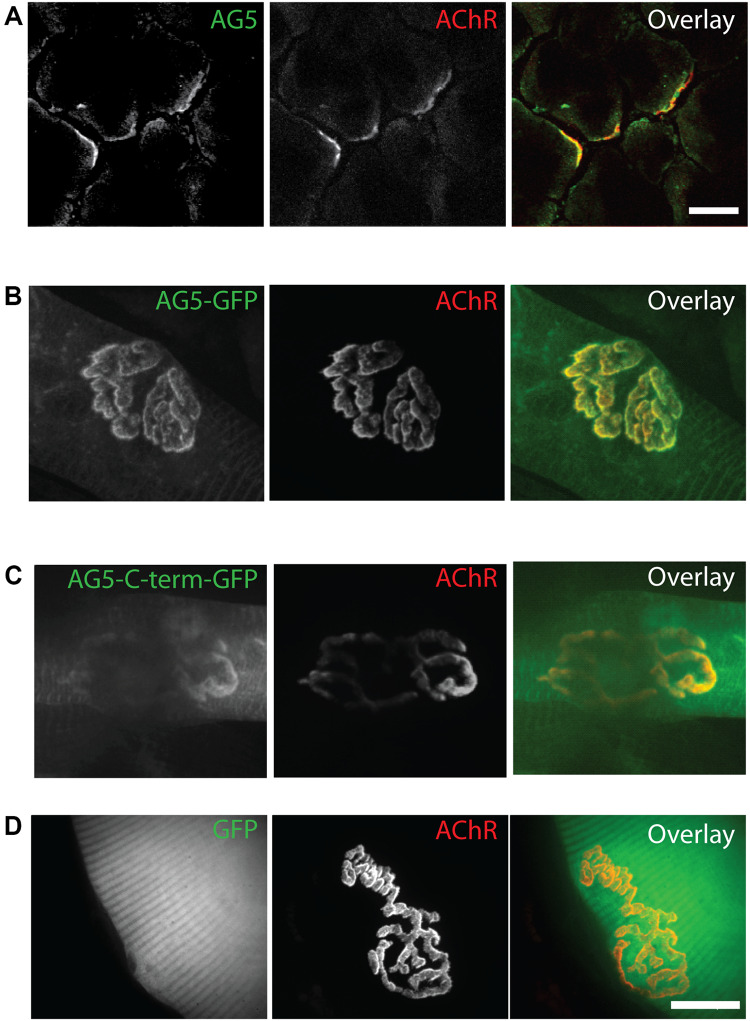
Arhgef5 localizes to AChR-rich regions of the NMJ. **(A)** Cryostat sections of TA muscle were analyzed by immunofluorescence. AChRs were detected using fluorescently labeled BTX. **(B–D)** TA muscle of P30 mice were electroporated with GFP-tagged Arhgef5 **(B)** or Arhgef5-C-term **(C)** or with GFP alone **(D)**. After 14 days, the muscle was fixed and stained with fluorescently labeled BTX. Single fibers were isolated and imaged. Scale bars are 20 μm.

α-dystrobrevin 1 and its phosphorylated form pY713-aDB1 concentrate in AChR-rich regions at the NMJ postsynaptic machinery (Grady et al., 2003; Gingras et al., 2016). We thus expected that Arhgef5 localizes in the same regions. Indeed, endogenous Arhgef5 colocalizes with AChR on the tibialis anterior (TA) muscle cross-sections (Figure 2A). To confirm independently that Arhgef5 is a novel synaptic protein, we electroporated muscles with plasmids expressing GFP-tagged Arhgef5 constructs. Both the longer and the shorter C-terminal isoform of Arhgef5 were enriched at the muscle postsynaptic machinery (Figures 2B,C). In contrast, overexpressed GFP (used as a control) did not concentrate at the NMJ (Figure 2D).

### Arhgef5 Loss Does Not Block AChR Cluster Formation in Cultured Myotubes

**FIGURE 3:**
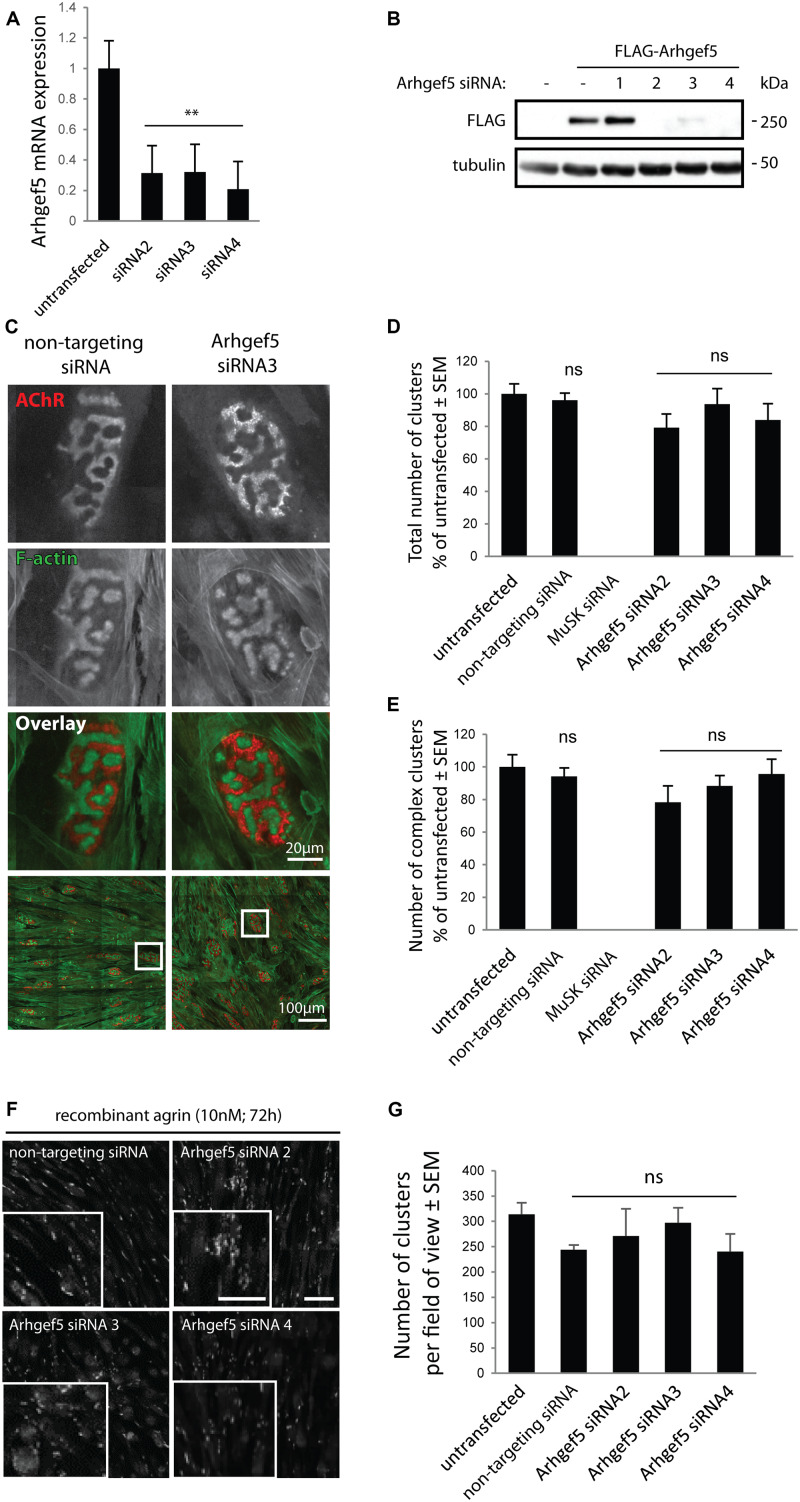
Arhgef5 is not required for AChR cluster formation in cultured myotubes. **(A)** C2C12 myotubes were transfected with control siRNA or three different siRNAs targeting Arhgef5. Arhgef5 mRNA levels were measured by RT-qPCR in the transfected cells. **(B)** HEK293T cells were transfected with FLAG-Arhgef5 and either control siRNA or four different siRNAs against Arhgef5 (siRNA 1, 2, 3, 4). FLAG-Arhgef5 levels were measured by Western blot. **(C–E)** C2C12 cells were transfected with negative control (non-targeting) siRNA, Arhgef5 siRNA, or MuSK siRNA as positive control. Cells were stained with fluorescently labeled BTX to visualize AChR and actistain to visualize F-actin. No differences in total cluster number or complex pretzel-like cluster formation was observed upon Arhgef5 knockdown. As expected, knockdown of MuSK fully abrogates AChR cluster formation. Scale bar is 20 μm or 100 μm, as indicated. **(F,G)** C2C12 were transfected as in **(C)** and treated with soluble agrin for 72 h. Cells were fixed and stained with fluorescently labeled BTX to visualize AChR. Arhgef5 knockdown does not affect the abundance of AChR cluster in cells stimulated with agrin. Scale bars are 100 μm. See Supplementary Figure S1 for an image of sparse AChR clusters that form in the absence of agrin or laminin. ^∗∗^*p* < 0.01.

Arhgef5 is an important regulator of podosome formation in MDCK and NIH/3T3 cells (Kuroiwa et al., 2011). Podosomes contribute to the formation of complex pretzel-like structures of the postsynaptic machinery in cultured myotubes (Proszynski et al., 2009), and aDB1-depleted myotubes fail to form complex AChR clusters (Pawlikowski and Maimone, 2009; Gingras et al., 2016). Accordingly, we predicted that inhibition of Arhgef5 function would impair complex cluster formation by blocking podosomes. To test this hypothesis, we knocked down the expression of Arhgef5 in C2C12 cells using siRNAs. Out of four siRNAs tested, three efficiently reduced Arhgef5 mRNA and protein levels (Figures 3A,B). Surprisingly, we found that Arhgef5 knock-down did not block either the appearance of pretzel-like postsynaptic machinery clusters in C2C12 cells cultured on laminin, or the formation of synaptic podosomes visualized by F-actin stained with phalloidin (Figures 3C–E). Moreover, Arhgef5 knock-down had no effect on the formation of simple AChR clusters that appear in C2C12 cells stimulated with agrin (Figures 3F,G).

### Generation of Mice With Muscle-Specific Arhgef5 Knockdown

**FIGURE 4:**
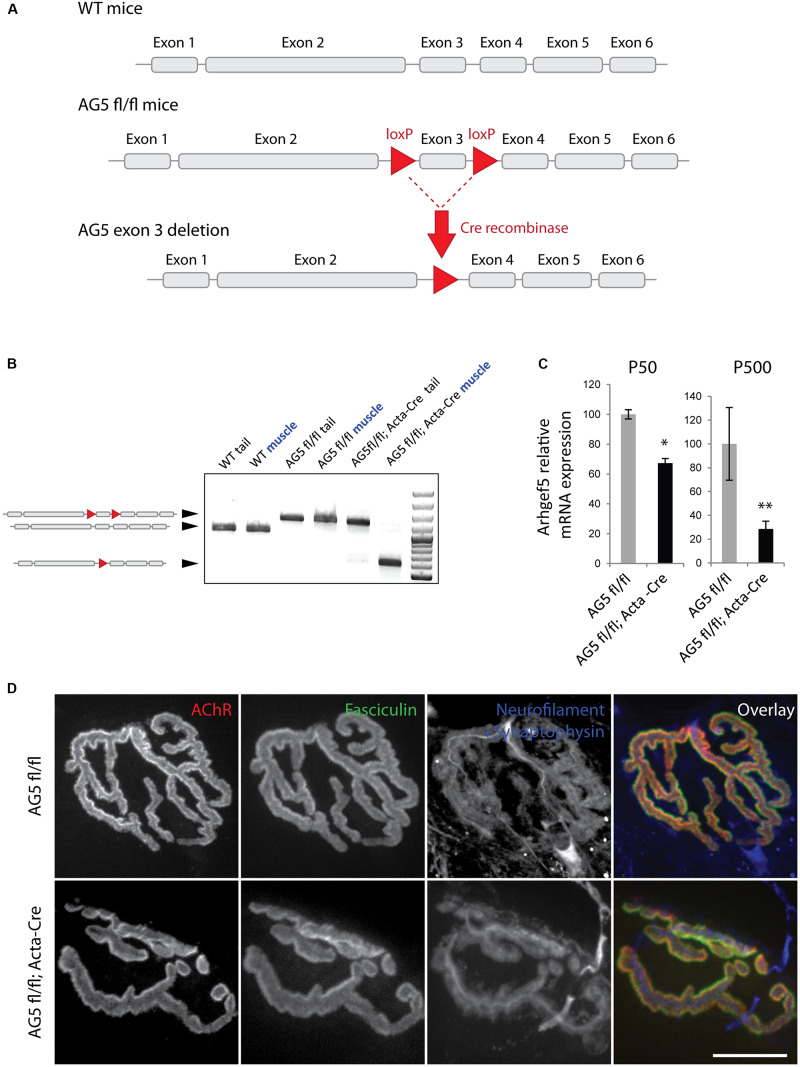
Generation of muscle-specific Arhgef5 KO mice. **(A)** Strategy for the generation of conditional Arhgef5 knockouts. Mice with loxP sites flanking the third exon of *Arhgef5* were crossed with Cre-recombinase expressing mice to produce knockout. **(B)** Genotyping shows successful recombination in the muscles but not tail tipss of AG5^*fl/fl*^; Acta-Cre mice. **(C)** Quantification of *Arhgef5* mRNA expression in AG5^*fl/fl*^ and AG5^*fl/fl*^; Acta-Cre mice at 50 and 500 days of age. Results are mean ± SEM of three mice from each genotype. **(D)** Proper alignment of pre- and post-synaptic elements in AG5^*fl/fl*^; Acta-Cre muscles. Single fibers of tibialis anterior muscles isolated from AG5^*fl/fl*^ and AG5^*fl/fl*^; Acta-Cre mice were stained with BTX (to visualize AChR), fasciculin II (to visualize the synaptic cleft marker acetylcholinesterase), and anti-neurofilament + anti-synaptophysin antibodies (to visualize the presynaptic nerve terminal). Scale bar is 20 μm. ^∗^*p* < 0.05, ^∗∗^*p* < 0.01.

The lack of a clear effect of *Arhgef5* knock-down on the postsynaptic machinery *in vitro* does not preclude the importance of Arhgef5 for NMJ formation or maintenance *in vivo*. We wanted to determine if depletion of Arhgef5 in mouse muscles would, like that of aDB1, cause abnormal NMJ morphology. To that end, we crossed *Arhgef5* flox/flox (AG5^*fl*/*fl*^) conditional knockout mice with mice expressing Cre under the control of the human skeletal actin (HAS or Acta) promoter, which is specifically activated in differentiated skeletal muscle fibers. We found that exon 3 of *Arhgef5* was excised in AG5^*fl*/*fl*^;Acta-Cre (Figures 4A,B), as evaluated by genotyping PCR reaction. We additionally measured the level of *Arhgef5* mRNA in muscles of AG5^*fl*/*fl*^;Acta-Cre and AG5^*fl*/*fl*^ mice. The expression of *Arhgef5* mRNA containing exon 3 in AG5^*fl*/*fl*^;Acta-Cre muscles was reduced compared to AG5^*fl*/*fl*^ mouse muscles, in which Cre was not expressed (Figure 4C). The expression went down over time from around postnatal day 50 (P50) to P500 with more apparent decline in older animals. The lack of complete elimination of *Arhgef5* expression in the muscles of AG5^*fl*/*fl*^;Acta-Cre mice could be attributed either to incomplete excision of all Arhgef5 alleles in the multinuclear myotubes or to contamination of samples with non-muscle cells, such as blood vessels, connective tissue, fibroblasts, and neurons. The decline of *Arhgef5* mRNA with age could also suggest that muscle fibers are supplemented with *Arhgef5* mRNA coming from muscle satellite cells, which are less numerous in older animals and in which the Acta promoter driving Cre expression is inactive. Because we could still detect residual *Arhgef5* in AG5^*fl*/*fl*^;Acta-Cre muscles, we refer to the genotype of these mice as muscle-specific *Arhgef5* knockdown rather than knockout.

### Neuromuscular Junctions Are Abnormally Fragmented in Arhgef5 Knockdown Muscles

To determine if the muscle-specific knockdown of Arhgef5 affects NMJ formation and maintenance, we isolated TA muscles from AG5^*fl*/*fl*^; Acta-Cre and AG5^*fl*/*fl*^ (control) mice and imaged their NMJs using α-bungarotoxin staining to visualize postsynaptic AChRs. In both types of mice NMJs formed normally with well aligned postsynaptic specialization (AChR), presynaptic elements (neurofilament and synaptophysin), and synaptic extracellular matrix marker (acetylcholinesterase stained with fasciculin II) (Figure 4D).

**FIGURE 5:**
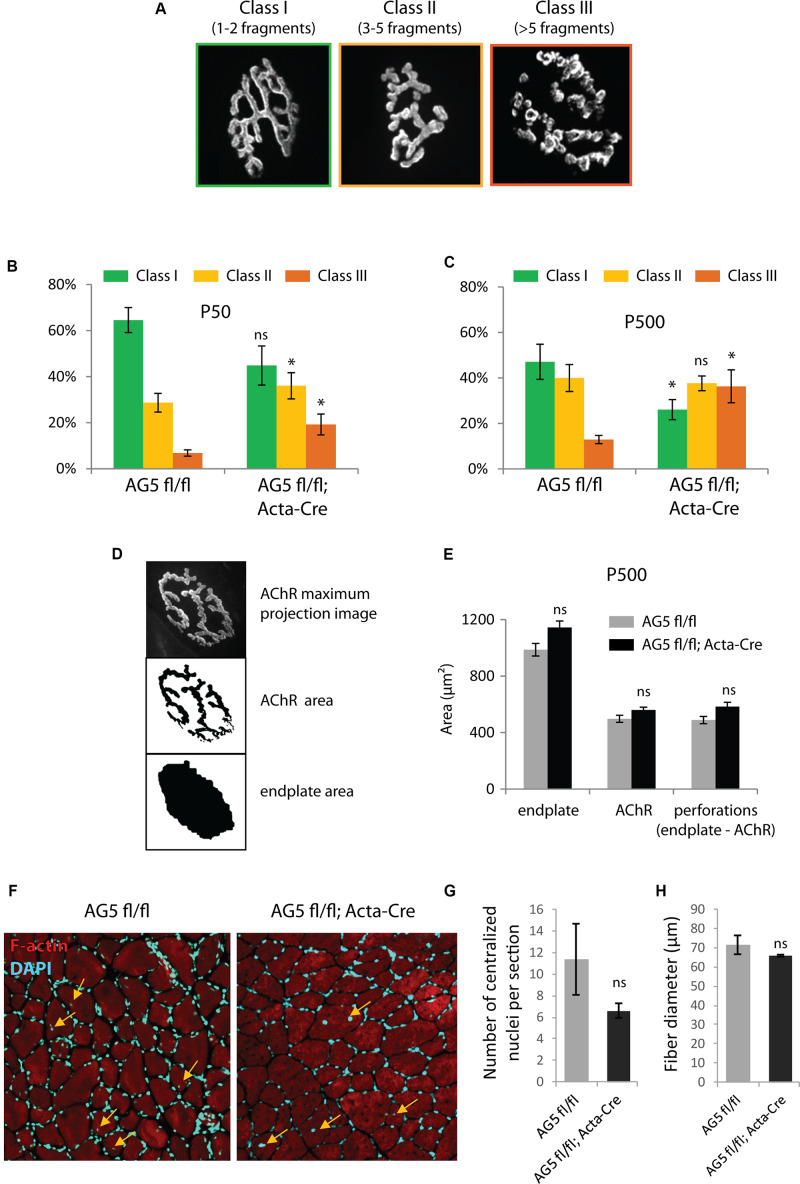
Muscle-specific loss of Arhgef5 results in progressive loss of NMJ integrity **(A)**. Classification of NMJs according to their fragmentation. Representative examples of NMJs from each class are shown **(B,C)**. Relative percentages of NMJs belonging to each of the three classes in tibialis anterior muscles of AG5^*fl/fl*^ and AG5^*fl/fl*^; Acta-Cre mice at 50 and 500 days of age. Results are mean ± SEM of three mice from each genotype. At least 30 synapses were counted for each mouse. Arhgef5 muscle-specific knocdown causes increased fragmentation of NMJs at both ages **(D)**. Visualization of the automated quantification of endplate and AChR areas of NMJs. The algorithm takes a maximum projection fluorescence image (top panel) and performs the segmentation of the image to calculate the AChR area (middle panel) or the total area of the endplate (bottom panel). Perforations area is calculated as the difference between endplate area and AChR area. **(E)** Neither the total endplate area nor the AChR area nor the perforations area were significantly different in tibialis anterior muscles of 500-day old AG5^*fl/fl*^; Acta-Cre mice compared to AG5^*fl/fl*^. Results are mean ± SEM. **(F)** Representative cross-sections of TA muscle of AG5^*fl/fl*^ and AG5^*fl/fl*^; Acta-Cre mice. F-actin was stained with Actistain and nuclei were stained with DAPI. Centralized nuclei are indicated with arrows. **(G)** Quantification of centralized nuclei per TA muscle cross-section for *n* = 3 mice per genotype. Results are mean ± SEM. **(H)** Quantification of fiber diameters in TA muscle cross-sections (samples as in **G**). Results are mean ± SEM. ^∗^*p* < 0.05.

Because the DGC is involved in the stability of the NMJ, we speculated that loss of Arhgef5 might result in more excessive fragmentation of NMJs. Indeed, when we scored NMJs according to the degree of fragmentation (Figure 5A), NMJs from AG5^*fl*/*fl*^;Acta-Cre showed more profound disintegration than control mice (Figure 5B). The effect of NMJ fragmentation was increased with age of animals and correlated with the decline in Arhgef5 mRNA (Figure 5C). This suggests that loss of Arhgef5 in muscles results in poorer NMJ stability.

To check if the increased NMJ fragmentation in mutant mice was associated with decreased total area of AChR-rich regions at each postsynaptic cluster, we performed unsupervised measurements of the total area of the NMJ, the area containing AChR receptors (α-bungarotoxin-positive) and the area of perforations between AChR-rich branches of the NMJ. Neither of these parameters was changed in AG5^*fl*/*fl*^;Acta-Cre mice compared to controls (Figures 5D,E).

We also wanted to make sure that the fragmentation of the postsynaptic machinery in AG5^*fl*/*fl*^;Acta-Cre mice is not a downstream consequence of increased degeneration and regeneration of muscle fibers. To that end, we measured the number of centralized nuclei per cross-section in TA muscles of AG5^*fl*/*fl*^;Acta-Cre mice and AG5^*fl*/*fl*^ controls. We did not observe increased numbers of centralized nuclei or abnormal fiber size in AG5^*fl*/*fl*^;Acta-Cre muscles, suggesting that the synaptic defects are not due to increased degeneration and regeneration of muscle fibers (Figures 5F–H and Supplementary Figure S2, Supplementary Movies S1–S3).

### Levels of Active Cdc42 and RhoA Are Reduced in Arhgef5 Knockdown Muscles

**FIGURE 6:**
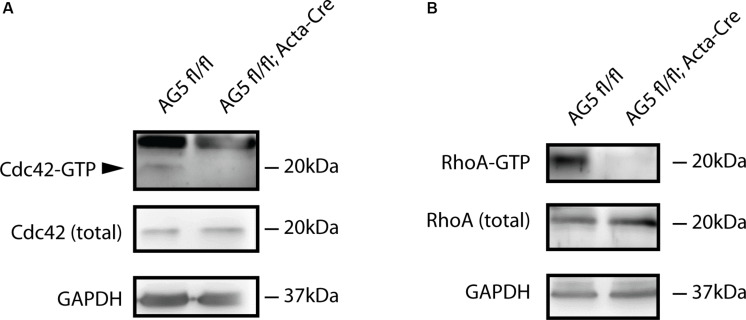
Active forms of Cdc42 and RhoA are reduced by muscle-specific Arhgef5 knockdown. **(A,B)** Active GTP-bound forms of Cdc42 **(A)** and RhoA **(B)** were precipitated from TA homogenates (upper panels) and Cdc42 and RhoA proteins were detected by Western blot from precipitates and tissue lysates (total). GAPDH was used as a loading control.

Arhgef5 is a guanidine exchange factor involved in the regulation of small GTPases from the Rho family. It activates these GTPases by inducing the exchange of GDP to GTP (Wang et al., 2009; Kuroiwa et al., 2011). Therefore, we reasoned that in Arhgef5-knockdown muscles activated GTP-bound Rho-family GTPases should be less abundant. Indeed, GTP-bound RhoA and Cdc42 were reduced in muscles from AG5^*fl*/*fl*^;Acta-Cre compared to control mice (Figure 6), suggesting that Arhgef5 is required for the maintenance of high levels of active RhoA and Cdc42, as previously shown in other cell types (Kuroiwa et al., 2011).

## Materials and Methods

### Plasmids

For co-IP Arhgef5 C-terminally tagged with a triple FLAG tag was cloned into pSBbi-Puro. GFP-aDB1 was engineered by cloning aDB1 cDNA into the pEGFP-C1 plasmid (Clontech) (Grady et al., 2003). For electroporation, full-length Arhgef5 or Arghef5 C-term were cloned into pCDNA3.1(+)/Puro in frame with the C-terminal GFP tag.

### Antibodies and Staining Reagents

The following antibodies were used for immunofluorescence: mouse anti-neurofilament (clone 2H3, Developmental Studies Hybridoma Bank), guinea pig anti-synaptophysin (Synaptic Systems). The following antibodies were used for western blot: rabbit anti-α tubulin (ab18251; Abcam), rabbit anti-GFP (GTX113617; Genetex), mouse anti-GFP (12A6; Developmental Studies Hybridoma Bank), mouse anti-GFP (9F9.F9; Novus Biologicals) mouse anti-FLAG (clone M2, F3165; Sigma), mouse anti-FLAG (635691; Clontech), mouse anti-β-actin (A1978; Sigma), mouse anti-α tubulin (T6199; Sigma). For visualization of AChR we used fluorescently labeled α-bungarotoxin (B35451 or B13422; Thermo), and for visualizing the synaptic cleft, we used fasciculin II, which binds to acetylcholinesterase (F-225; Alomone Labs, labeled with FluoReporter FITC kit from Thermo).

### Western Blot

SDS-PAGE was performed on hand-cast polyacrylamide gels. Transfer was performed using *Trans-*Blot Turbo (Bio-Rad) onto nitrocellulose membranes (0.2 μm pore size; PALL). For detection, we used HRP-coupled secondary antibodies (Jackson) and Clarity Western ECL substrate (Bio-Rad). ECL images were collected on photographic film (CL-XPosure, Thermo) and digitized using a film scanner (Epson) or were digitally acquired on the Amersham 680RGB instrument.

### Peptide Pulldown

Pulldown of Arhgef5 using synthetic peptides was performed as in Gingras et al. (2016). Briefly, synthetic peptides (phospho-Y713: DEST–Biotin–Ahx–TQPEDGNpYENESVRQ-NH2, Y713: DEST–Biotin–Ahx–TQPEDGNYENESVRQ-NH2) were purch- ased from Lifetein. The peptides were bound to Streptavidin-coupled Dynabeads M-280 (Life Technologies). Recombinant Arhgef5-GST (C-terminal; Proteintech) was resuspended in bead binding buffer [20 mM Hepes, 150 mM NaCl, 0.5 mM EDTA, 10% Glycerol, 0.05% Nonidet P-40, 1 mM DTT, EDTA-free Mini protease inhibitor cocktail (Roche), pH 7.9] and incubated with the beads overnight at 4°C. The beads were washed 2x in bead binding buffer, and the proteins were eluted in 2x Laemmli sample buffer with 50 mM DTT for 10 min at 95°C.

### Co-immunoprecipitation

HEK 293T cells were transfected with plasmids encoding α-dystrobrevin 1-GFP, Arhgef5-FLAG, or both for 24 h. Cells were lysed in lysis buffer: 50 mM Tris, 150 mM NaCl, 0.1% Nonidet P-40, 50 mM NaH2PO4, 1 mM sodium pervanadate, 10 mM NaF, 1x SigmaFAST EDTA-free protease inhibitors (Sigma), 2 μM bortezomib and incubated with Dynabeads-protein G (Life Technologies) precoated with rabbit anti-GFP (GTX113617; Genetex) for 1 h at 4°C or with rabbit-anti-FLAG (PA1984B; Thermo) or rabbit IgG isotype control (Sigma) overnight at 4°C. Beads were washed 3x with lysis buffer and protein was eluted using 2x Laemmli sample buffer for 30 min at 65°C.

### Animals

All animals used in the study were on the C57/Bl6 genetic background. Acta-Cre mice (Jax #: 006149; B6.Cg-Tg[ACTA1-cre]79Jme/J) were purchased from The Jackson Laboratory (Stock No: 006149) (Miniou et al., 1999). Arhgef5 conditional knockout mouse line (C57BL/6N-Arhgef5 < tm1c(NCOM)Mfgc > /Tcp) was generated at the Mammalian Functional Genomics Centre, University of Manitoba and provided through The Canadian Mouse Mutant Repository at The Hospital for Sick Children (Toronto, ON, Canada) (Bradley et al., 2012). The original allele was created under the North American Conditional Mouse Mutagenesis (NorCOMM) Project. Animals were kept at the Nencki Institute Animal House on a 12 h light/dark cycle with food and water *ad libitum*. All experiments were conducted in accordance with the animal experimentation permit from the local ethics committee.

### Genotyping

Genomic DNA was isolated from mouse tail tips or TA muscles using Chelex 100 (Sigma). A small piece of tissue was added into 200 μl of 10% Chelex solution, incubated at 95°C for 20 min, and centrifuged at 12 000 *g* at RT for 10 min. The supernatant was collected and used for the PCR reactions as a template. PCR was performed using 2 × PCR Master Mix (Thermo) using the following primers: CACCTACATCAGCTCAGAAAGTCAT (forward), CAACAGGTGACTCTGACACAGCA (reverse).

### RNA Isolation and qRT-PCR

RNA was isolated using TRIsure reagent (Bioline), and cDNA was synthesized using a High-Capacity cDNA Reverse Transcription Kit (Thermo) according to the manufacturer’s instructions. qRT-PCR was performed using the Step One Plus Real-Time PCR system (Thermo) and SYBR Green PCR Master Mix (Thermo). The instrument’s default programs were used for data acquisition, and the results were analyzed using the comparative Ct method for relative quantification. The housekeeping gene glyceraldehyde-3-phosphate dehydrogenase (*Gapdh*) was used to standardize the samples. The following primers were used: *Gapdh* (GGCCTTCCGTGTTCCTAC and TGTCATCATACTTGGCAGGTT) and *Arhgef5* (CCAA CAAGCAAAAGGGCTGG and TCTTCTCCCGGAAAACAA CAGA).

### Electroporation of TA Muscle

Mice were anesthetized using intraperitoneal injection of ketamine (90 mg/kg) and xylazine (4.5 mg/kg). Five to 10 min after the addition of anesthetics, the leg was shaved using hair removal cream. 25 μl of ∼1 mg/mL solution of DNA were injected into the TA muscle with a Hamilton syringe followed by electroporation of the whole leg 10 × with 20 ms pulses at 1 Hz by BTX ECM 830 square wave electroporator. Electrodes were wetted with PBS to enable ion flow and the voltage was ∼175 V/cm. Postoperative analgesia was achieved by subcutaneous injection of 1–5 mg/kg of butorphanol and local anesthesia using 2% lidocaine gel. Mice were sacrificed by pentobarbital injection 14 days after the procedure.

### Fresh-Frozen Tissue Sections Immunofluorescence

Tibialis anterior muscle was frozen in cold isopentane and sectioned on a cryostat. Slides were stained with an anti-Arhgef5 antibody and α-bungarotoxin.

### Fixed Muscle Fiber Immunostaining and Imaging

Immediately after sacrificing the animal, the skin of the leg was surgically removed and fascia from the TA muscle was taken off. The whole leg was severed and incubated in fresh 4% PFA for 1 h at room temperature. The leg was washed 3 × 5 min in PBS and stored in PBS containing 0.02% sodium azide at 4°C. Fixed muscles were teased into single fibers using fine forceps. Non-specific staining was blocked with 2% bovine serum albumin (BSA) and 2% goat serum in PBS supplemented with 0.5% Triton-X100. The fibers were incubated with primary antibodies overnight in the same buffer. Fibers were washed 3 × 5 min in PBS followed by incubation with secondary antibodies and fluorescently labeled bungarotoxin in 2% BSA and 2% goat serum in PBS containing 0.1% Triton-X100 for 1 h at room temperature. Cells were washed 3 × 5 min in PBS and mounted in fluoromount with DAPI, covered with coverslips, and sealed with nail polish. Image acquisition was performed at the Confocal Microscopy Facility at the Nencki Institute, using the Zeiss spinning disk confocal microscope or the Leica TCS SP8 scanning confocal microscope. Images were analyzed using ImageJ/Fiji software.

### NMJ Phenotype Quantification

Tibialis anterior muscles were fixed in 4% PFA and stained with fluorescently labeled bungarotoxin. Teased fibers from the muscle were mounted on slides and imaged using the Zeiss spinning disk confocal microscope. To ensure reliable measurements, only NMJs that were placed directly facing and parallel to the coverslip were used for downstream analyses. NMJs (30–50 per animal) were manually assigned to one of three populations based on the number of discrete fragments they were composed of: class I contained 1-2 fragments, class II contained 3-5 fragments and class III contained 6 fragments or more. For morphometric analysis, we used the “NMJ-morph” ImageJ plugin (Jones et al., 2016).

### Quantification of Centralized Nuclei

Tibialis anterior muscles were dissected, embedded in OCT and snap-frozen in isopentane cooled by liquid nitrogen and stored at −80°C at least overnight. Transverse 20 μm-thick sections were generated by using Leica Cryostat CM1950, collected on slides, dried at room temperature for 1 h and stored at −20°C. For immunohistochemistry, sections were fixed with 4% PFA for 10 min at room temperature and then treated with blocking solution for 1 h [0.2% BSA, 0.5% Triton X-100, 2% normal goat serum (NGS) in phosphate-buffered saline (PBS)]. Sections were counterstained with AlexaFluor-555-conjugated phalloidin (A34055, Thermo Scientific) at 1:500 dilution in blocking solution for 1 h at room temperature. Afterward, slides were washed for 5 min in PBS. Coverslips were applied over the slides and mounted with mounting media containing 1:1000 dilution of DAPI (A4099, AppliChem). Immunofluorescence microscopy was performed using a spinning disk confocal microscope (Zeiss).

### Cell Culture

C2C12 myoblasts were cultured as described previously (Pęziński et al., 2019). Briefly, myoblasts were cultured on 0.2% gelatin-coated plates in Dulbecco’s Modified Eagle Medium (DMEM) supplemented with 20% fetal bovine serum (FBS), 4.5 g/L glucose, L-glutamine, penicillin, streptomycin, and fungizone. For myotube fusion, cells were trypsinized and plated on Permanox slides (VWR) in eight-well Flexiperm chambers (Sarstedt). The slides were coated with 111-laminin (Sigma). To induce myotube differentiation, the media were replaced with DMEM with 2% horse serum. For experiments involving agrin, slides were coated with 0.2% gelatin instead of laminin, and myotubes were stimulated with 10 nM recombinant rat Z’-spliced agrin (R&D Systems) for 72 h. The cells were fixed in 4% paraformaldehyde (PFA) and permeabilized with 0.5% Triton X-100 for 30 min. The myotubes were stained using Alexa Fluor 555-coupled bugarotoxin (BTX) (B35451, Life Technologies) and Acti-Stain 488 (PHDG1, Cytoskeleton). Cells were imaged on the Zeiss spinning disk confocal microscope at the Nencki Institute Microscopy Core Facility. Images were processed using ImageJ/Fiji and analyzed in R.

### Arhgef5 Knock Down

Arhgef5 silencing in differentiated C2C12 myotubes was performed with Lipofectamine RNAiMAX (Life Technologies). The following siRNAs were used at 20 nM: siRNA1 (#2430, Sigma-Aldrich), siRNA2 (#30088, Sigma-Aldrich), siRNA3 (#6911, Sigma-Aldrich), siRNA4 (#4390771, ID: s79541, Ambion), and non-targeting control siRNA (#12935300, Invitrogen). The cells were fixed 2 days later and examined by confocal microscopy.

### RhoA/Cdc42 Activity Assay

Experiments were carried out as described previously with minor modifications (Bijata et al., 2015; Schill et al., 2020). Briefly, for biochemical determination of RhoA/Cdc42 activity TA muscles were homogenized in lysis buffer [25 mM HEPES (pH 7.5), 150 mM NaCl, 1% Nonidet P-40, 10 mM MgCl2, 1 mM ethylenediaminetetraacetic acid (EDTA), and 2% glycerol] and centrifuged at 14000 × *g* for 10 min at 4°C. The extracts were incubated with GST-PAK-PBD fusion protein that had been conjugated with glutathione beads (for Cdc42; Cell BioLabs) at 4°C overnight or an anti-active RhoA monoclonal antibody and protein A/G Agarose beads (for RhoA, Neweast Biosciences) for 1 h at 4°C and then washed three times with lysis buffer. Active RhoA/Cdc42 was analyzed by SDS-PAGE and subsequently immunoblotted with RhoA-specific antibody (Neweast Biosciences, 1:500) and Cdc42-specific antibody (11A11, Cell Signaling, 1:500).

### Statistics

Statistical analyses were performed using GraphPad Prism 7, Microsoft Excel, and R. Quantitative data are presented as the mean ± SEM or SD. Two-tailed unpaired *t*-test or ANOVA with Bonferroni’s *post hoc* test were used to test for statistical significance, as appropriate. *P* > 0.05 was labeled as ns-not significant, *p* < 0.05 as ^∗^, *p* < 0.01 as ^∗∗^, *p* < 0.001 as ^∗∗∗^, and *p* < 0.0001 as ^****^. Values of *p* < 0.05 were considered statistically significant.

## Discussion

The DGC is essential for the maturation and maintenance of the NMJ. The dysfunctions of its components often lead to myopathies, such as Duchenne muscular dystrophy or limb-girdle muscular dystrophy (Mercuri et al., 2019). One of the DGC components is aDB1, whose deletion in mice results in lower density, decreased stability and abnormal distribution of AChR at the NMJ (Grady et al., 2003, 2000; Pawlikowski and Maimone, 2009). It has been recently proposed that aDB1 serves as a scaffold for the binding of signaling molecules, including Liprin-α1 (Bernadzki et al., 2017), Grb2, α-catulin (Lyssand et al., 2010; Gingras et al., 2016), and the Rho GEF Arhgef2. Here, we showed that phospho-aDB1-binding protein Arhgef5 accumulates at the NMJ and is required for the maintenance of NMJ integrity *in vivo*. Arhgef5 was of particular interest to us, because it had previously found to bind another aDB1 interactor α-catulin in HEK293 cells (Lyssand et al., 2010), and because it is known to be essential for the formation of Src-induced podosomes in fibroblasts (Kuroiwa et al., 2011). We therefore hypothesized that Arhgef5 promotes the remodeling of NMJ postsynaptic machinery through actin dynamics and synaptic podosomes in muscle cells. However, the knockdown of Arhgef5 failed to affect the formation of synaptic podosomes in cultured myotubes and had no obvious effect on the formation of AChR clusters. The most parsimonious explanation for that result is that C2C12 cells, unlike fibroblasts, do not rely on Arhgef5 to activate Rho family GTPases and induce podosomes. Two potential candidates for such a function in C2C12 cells are RhoGEFs ephexin1 and Arhgef2. We have previously shown that Arhgef2 also associates with phospho-aDB1, suggesting that it could be compensating for the loss of Arhgef5 in C2C12 cells (Gingras et al., 2016). Ephexin1 had been shown to affect the maturation of postsynaptic clusters in C2C12 cells (Shi et al., 2010) and NMJ *in vivo*, but whether it does so in collaboration with the DGC or through the regulation of actin-rich podosomes remains unknown.

In contrast to the apparent lack of effect of Arhgef5 loss in cultured myotubes, the knockdown of Arhgef5 in muscle fibers *in vivo* leads to synaptic abnormalities. NMJs in muscle-specific Arhgef5 KO mice are more fragmented, implying that Arhgef5 is important for NMJ stability once the postsynaptic maturation is accomplished. This phenotype does not mimic the abnormalities observed in aDB1 KO mice, whose NMJs lack a clear demarcation between AChR-rich and AChR-poor areas, resulting in a “fuzzy” appearance (Grady et al., 2000; Wang et al., 2018). Since aDB1 recruits many signaling proteins, it is plausible that the synaptic defects in the absence of aDB1 and Arhgef5 differ. On the other hand, Arhgef5 becomes depleted in conditional KO muscles only later in life (Figure 4C), so it is possible that the observed differences between the phenotypes of aDB1 KO and Arhgef5 muscle-specific knockdown mice are due at least in part to the different timing of the loss of function of their respective genes. Therefore, it is difficult to speculate about the lack of Arhgef5 function in synaptic development.

The knockout of another RhoGEF ephexin1 has also been shown to cause fragmentation of the NMJ postsynaptic machinery (Shi et al., 2010). However, the mechanisms of action of ephexin1 and Arhgef5 appear to be different. Whereas ephexin1 plays a pivotal role in the early development of NMJs and the creation of complex postsynaptic pretzels *in vivo*, loss of Arhgef5 in the muscle does not induce early developmental aberrations of the NMJ (data not shown). It is only later in development that the effects of Arhgef5 knockdown become more pronounced. This could explain why depletion of Arhgef5 has no effect on the short-lived AChR clusters in C2C12 cells. However, as discussed above, Arhgef5 only becomes strongly depleted in adult conditional KO mice, so it is plausible that an earlier loss of function of Arhgef5 might result in a different phenotype more reminiscent of ephexin1 KO mice.

How might Arhgef5 affect the stability of NMJs *in vivo*? Our data indicate that the mechanism might involve the regulation of cytoskeleton dynamics via small Rho-family GTPases. We showed that the active GTP-bound forms of RhoA and Cdc42 are reduced in the muscles of conditional AG5 KO mice. Reduced RhoA activity was shown to be the main causal factor in the abnormal NMJ phenotype of epehexin1 KO mice (Shi et al., 2010). Moreover, RhoA is essential for the fusion of microclusters of AChR into larger clusters in muscle cells (Weston et al., 2003), which might explain the loss of integrity of NMJs in AG5 conditional KO mice. RhoA may also help anchor the NMJ to the cytoskeleton by promoting the expression and membrane localization of utrophin, a DGC component known to bind actin filaments via its N-terminal tail (Galkin et al., 2002; Bonet-Kerrache et al., 2005). Cdc42 is also important in the formation of AChR clusters in mammalian muscle cells (Weston et al., 2000) and for the proper formation of the NMJ postsynaptic machinery in Drosophila (Rodal et al., 2008). In conclusion, it appears that Rho GEFs and their target small GTPases are important players in the formation, maturation, and maintenance of the postsynaptic structures of the NMJ. Going forward, it will be important to decipher the respective roles of the different GEFs at various time points of NMJ development, and their potential involvement in muscular dystrophies in humans.

## Data Availability Statement

All datasets generated for this study are included in the article/Supplementary Material.

## Ethics Statement

The animal study was reviewed and approved by the 1st Local Ethics Committee for Animal Experiments in Warsaw.

## Author Contributions

TP and PN devised the study and edited the manuscript. KB, PD, KR, MP, MG, BP, TC, MB, KB, TP, and PN designed and performed the experiments and analyzed the data. JW, TP, and PN supervised the study. PN, KB, PD, KR, MG, TC, and MB wrote sections of the first draft of the manuscript.

## Conflict of Interest

The authors declare that the research was conducted in the absence of any commercial or financial relationships that could be construed as a potential conflict of interest.

## Supplementary Material

FIGURE S1 Image of spontaneous clusters of AChR that form in C2C12 myotubes in the absence of agrin and laminin. AChR was labeled with fluorescently labeled BTX.

FIGURE S2 Images of fragmented synapses corresponding to X-Z cross-sections shown in Supplementary Movies S1–S3. AChRs were stained with fluorescently labeled BTX (green). Nuclei were stained with DAPI (magenta). Maximum projection images through the fibers are shown.

MOVIES S1–S3 X-Z slices through TA muscle fibers with fragmented NMJs in AG5^*fl/fl*^; Acta-Cre mice. We did not see any evidence of increased abundance of centralized nuclei in these fibers. AChRs were stained with fluorescently labeled BTX (green). Nuclei were stained with DAPI (magenta).
