# Supplementary figures and images for: Arhgef5 Binds α-Dystrobrevin 1 and Regulates Neuromuscular Junction Integrity
Source: Front Mol Neurosci. 2020 Jun 10;13:104. doi: 10.3389/fnmol.2020.00104 (PMC7299196; doi:10.3389/fnmol.2020.00104)

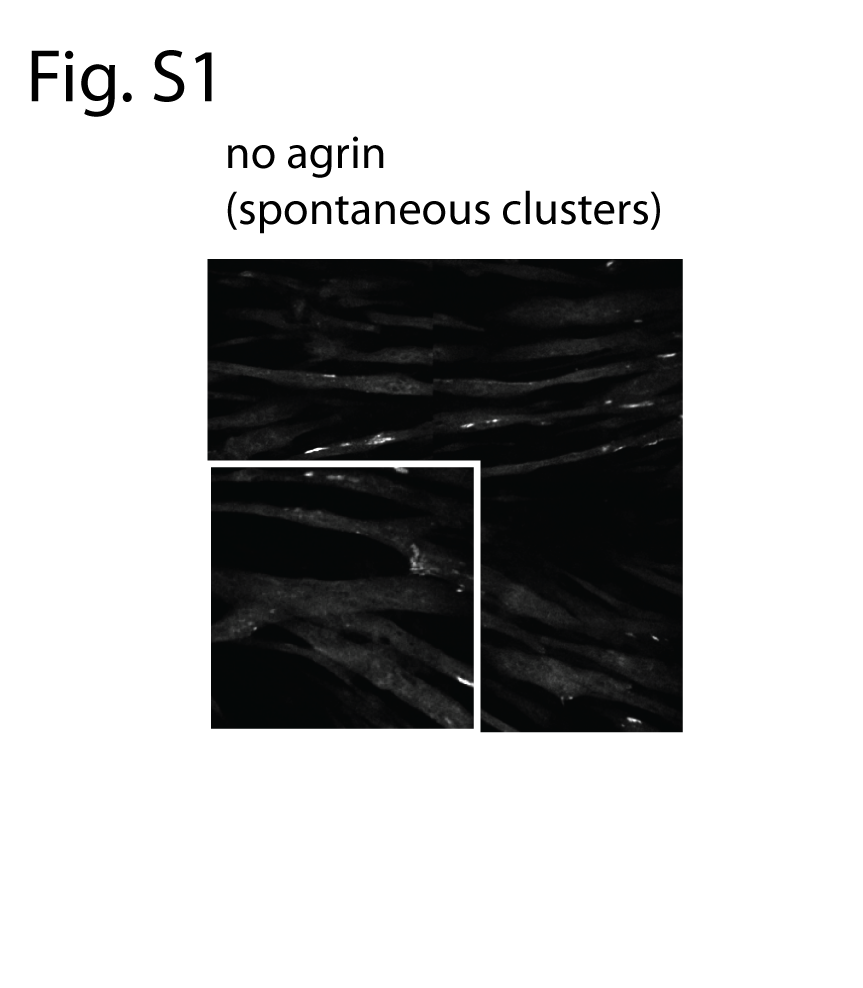

Supplement: FIGURE S1 — Image of spontaneous clusters of AChR that form in C2C12 myotubes in the absence of agrin and laminin. AChR was labeled with fluorescently labeled BTX. [file Image_1.TIF]

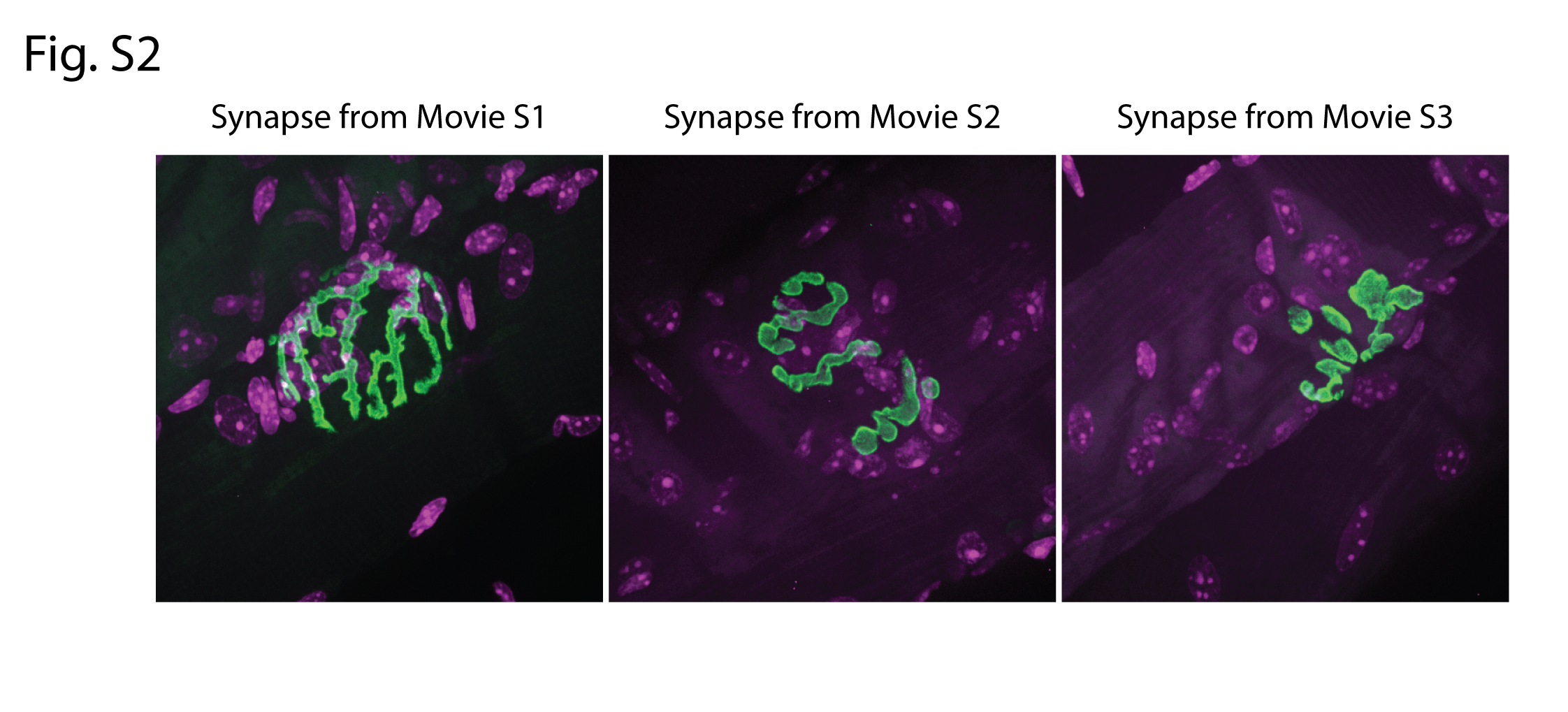

Supplement: FIGURE S2 — Images of fragmented synapses corresponding to X-Z cross-sections shown in Supplementary Movies S1–S3. AChRs were stained with fluorescently labeled BTX (green). Nuclei were stained with DAPI (magenta). Maximum projection images through the fibers are shown. [file Image_2.TIF]
